# Biological and Clinicopathological Characteristics of OPN in Cervical Cancers

**DOI:** 10.3389/fgene.2022.836509

**Published:** 2022-05-20

**Authors:** Shuhang Qin, Li Yi, Yanchun Liang, Yili Chen, Wei Wang, Yuandong Liao, Chunyu Zhang, Hua Huang, Jiaming Huang, Shuzhong Yao

**Affiliations:** ^1^ Department of Obstetrics and Gynecology, The First Affiliated Hospital, Sun Yat-sen University, Guangzhou, China; ^2^ Department of Neurosurgery, Union Hospital, Tongji Medical College, Huazhong University of Science and Technology, Wuhan, China

**Keywords:** osteopontin, cervical cancer, immunosuppression, biological process, biomarker

## Abstract

**Background:** Cervical cancer (CC) is the most common gynecological malignancy. Recently, an increasing number of studies have indicated that osteopontin (OPN) is a promising diagnostic and prognostic biomarker for CC. However, the biological role and detailed mechanism of OPN in CC remain unclear.

**Methods:** The Cancer Genome Atlas (TCGA) and Gene Expression Omnibus (GEO) datasets and a clinical sample microarray were used in our study. To identify the clinicopathological characteristics of OPN in CC, we compared the expression of OPN between normal and CC tissue samples and analyzed the correlations between OPN expression and multiple clinicopathological features. To identify biological processes involving OPN, OPN-associated genes were screened with Pearson correlation analysis and applied in hallmark gene set enrichment analysis (GSEA). Additionally, leukocyte infiltration was evaluated based on OPN expression. Finally, OPN-related signaling pathways were identified by GSEA.

**Results:** OPN expression was higher in CC samples than in normal tissue samples and positively correlated with age, FIGO stage, tumor size, lymphovascular invasion and an unfavorable prognosis. OPN-associated genes were mainly enriched in the immune response, and increased OPN expression was accompanied by increased M2 macrophage infiltration. Additionally, OPN was correlated with hypoxia, high glycolytic metabolism, apoptosis, angiogenesis, epithelial-mesenchymal transition and multiple signaling pathways (the p53 pathway, the PI3K/Akt pathway, IL6/STAT3 signaling, mTORC1 signaling and KRAS signaling).

**Conclusion:** Our study showed that OPN is involved in immunological activities and multiple tumor processes, identifying it as a potential therapeutic target and useful prognostic factor in CC patients.

## Introduction

As the most common gynecological malignancy, uterine cervical cancer (CC) is the fourth most frequent female cancer worldwide. According to global cancer statistics, there were approximately 570,000 new CC cases and 311,000 deaths worldwide in 2018 (Bray et al., 2018). Although major achievements have been made in surgery, chemotherapy, and radiotherapy in recent decades, these therapeutic approaches can hardly prevent metastasis and recurrence in CC patients. As a new therapeutic strategy, molecular targeted therapy is urgently required. It is important to identify key prognostic factors and predictive biomarkers to improve the effectiveness of treatment and develop precise treatment strategies.

Uterine CC is primarily caused by infection with high-risk human papillomavirus (HPV). Moreover, the tumor microenvironment plays a significant role in the tumorigenesis of CC. Numerous studies have identified immunity- or metabolism-related gene signatures as prognostic biomarkers in a wide spectrum of human malignancies (Zhang et al., 2003; Galon et al., 2006; Mahmoud et al., 2011). Osteopontin (OPN), known previously as secreted phosphoprotein 1 (SPP1), is a small integrin-binding ligand N-linked glycoprotein that binds to cell-surface receptors, including integrins and CD44 (Oldberg et al., 1986; Thoms et al., 2012). OPN is commonly found in some mineralized tissues as an extracellular matrix component, and it also behaves as a secreted protein in body fluids, such as milk, blood, urine, saliva, seminal fluid and bile. As a result of its diverse distribution, OPN is involved in multiple characteristics of tumor biology, including cell proliferation, survival, angiogenesis, chemoresistance, stem-like properties, tumor invasion, and metastasis (Wei et al., 2017). Furthermore, recent studies have shown that OPN plays important roles in antitumor immunity and metabolism (Wang and Denhardt, 2008; Hsieh et al., 2014; Shi et al., 2014; Moorman et al., 2020). As an immunological and metabolic biomarker, OPN might participate in the progression of CC.

In previous reports, OPN was found to act as an oncogene during tumorigenesis of CC (Cho et al., 2008; Bao et al., 2015; Xu et al., 2019). High expression of OPN was correlated with a higher FIGO stage, larger tumor size, lymph node metastasis, HPV infection, cisplatin resistance and shorter survival (Bao et al., 2015; Huang et al., 2015; Xu et al., 2019). Nonetheless, the immunological and metabolic features of OPN in CC have not been sufficiently investigated, and clinical characteristic analysis using gene datasets from multiple databases has never been performed. Given that OPN could be a promising target in CC, comprehensive reports of the relationships between OPN gene expression and clinical outcomes or biological features in CC are still required.

In this study, we collected a dataset from The Cancer Genome Atlas (TCGA), eight Gene Expression Omnibus (GEO) datasets and our clinical sample microarray to explore the potential role of OPN in CC. First, we compared OPN gene expression between CC and normal tissues. Second, we analyzed the correlations between OPN expression and clinical characteristics. Finally, we explored the potential biological processes and signaling pathways involving OPN in the progression of CC. This is the first integrative bioinformatic analysis to characterize OPN expression in CC molecularly and clinically and provide novel insight to improve the comprehensive understanding of the molecular mechanism of OPN in CC development.

## Materials and Methods

### Datasets

Normalized TCGA gene expression data and clinicopathological data (304 CC patients) were downloaded from the University of California Santa Cruz (UCSC) Xena project (https://xenabrowser.net/datapages/) (Supplementary Table S1). According to the UCSC pipeline, gene expression data is re-computed by log2 (fpkm-uq+1) and the fpkm-uq value is a modified version of the fpkm formula (normalized expression value). The comparison of OPN expression between CC and nontumor tissues was performed by Gene Expression Profiling Interactive Analysis (GEPIA; http://gepia.cancer-pku.cn/). The microarray datasets (GSE39001, GSE9750, GSE7803, GSE63514, GSE27678, GSE67522, GSE46857 and GSE44001) were downloaded from GEO series_matrix data (https://www.ncbi.nlm.nih.gov/geo/) and were normalized in the R statistical environment using the affy Bioconductor library NormalizeBetweenArrays and Log2 transformation. (Table 1, Supplementary Table S2).

**TABLE 1:** Clinical properties of the CC patients used in the GEO datasets analysis.

GEO ID	Contributors	GEO Platform	No. of Normal Samples in Dataset	No. of Tumor Samples in Dataset	No. of Squamous Intraepithelial Lesion (HSIL/LSIL)^a^	No. of Cervical Squamous Intraepithelial Lesion (CIN1/CIN2/CIN3)^b^
GSE39001	Espinosa AM et al., 2013	GPL201	12	43	—	—
GSE9750	Murty VV et al., 2008	GPL96	24	33	—	—
GSE7803	Zhai Y et al., 2007	GPL96	10	21	HSIL: 7	—
GSE63514	Den Boon J et al., 2015	GPL570	24	28	—	CIN1: 14; CIN2: 22; CIN3: 40
GSE27678	Karagavriilidou K et al., 2013	GPL571	12	—	HSIL: 21; LSIL: 11	—
GSE67522	Sharma S et al., 2015	GPL10558	22	20	—	—
GSE46857	Mulherkar R et al., 2013	GPL7025	4	25	—	—
GSE44001	Lee YY et al., 2013	GPL14951	—	300	—	—

a HSIL: high-grade squamous intraepithelial lesion; LSIL: low-grade squamous intraepithelial lesion.

b CIN: cervical squamous intraepithelial lesion.

### Clinical Sample Microarray Sequencing

Microarray data for five CC samples and six normal cervical tissue samples from the Department of Gynecology, First Affiliated Hospital of Sun Yat-sen University were used to compare the expression of OPN between CC and nontumor tissues. Sample collection, RNA sequencing and data normalization have been described previously (Huang et al., 2018). Written informed consent was obtained from each patient. All specimens were handled according to legal and ethical standards.

### OPN-associated gene identification and Hallmark gene set enrichment analysis (GSEA)

The Pearson correlation coefficients for OPN with all other protein-coding genes were calculated in RStudio Version 1.2.1335 (R Version 3.6.3) with the cor. test algorithm. Genes with an R value >0.2 and a *p* value < 0.05 were defined as the threshold for OPN-associated genes in each dataset. All the OPN-associated genes were introduced into the Metascape website (http://metascape.org/gp/index.html) for Hallmark GSEA. The hallmark gene set V7.2 from the Broad Institute (http://software.broadinstitute.org/gsea/msigdb/collections.jsp#H) contains specific well-defined biological states and processes and displays coherent expression (Liberzon et al., 2015).

The correlations of OPN with immune checkpoint molecules, hypoxia-related genes and glycolytic enzymes were visualized with Circos plots produced with R software using the “circlize” package. The correlations of OPN with apoptosis, angiogenesis and epithelial-mesenchymal transition (EMT) were visualized with a correlogram using the “corrplot” package. Hypoxia-related genes and biomarkers of apoptosis and angiogenesis were selected from the Hallmark gene set.

### Tumor Purity and Leukocyte Infiltration

The ESTIMATE tool (https://bioinformatics.mdanderson.org/estimate/disease.html) was used to analyze tumor purity. The CIBERSORT tool (https://cibersort.stanford.edu/) was used to evaluate leukocyte infiltration. The correlations of OPN with tumor purity and leukocyte infiltration were calculated with Pearson correlation analysis and are shown in heatmaps produced by MORPHEUS (https://software.broadinstitute.org/morpheus/). The samples were ordered according to the expression of OPN.

### Pathway Gene Signatures Analyzed Using GSEA

GSEA was used to compare dysfunctional pathways between CC samples with high OPN expression and those with low expression in the TCGA. GSEA software (version: 4.0.3) and the Hallmark gene set V7.2 were applied. The upregulated pathways were defined by a normalized enrichment score (NES) > 0 and are listed in Supplementary Table S5. Pathways with a normalized *p* value <0.05 were considered significantly enriched.

### Statistical Analyses

An unpaired *t* test was used to compare OPN expression between two groups in TCGA, GEO datasets and clinical microarray data. An unpaired *t* test was used to compare OPN expression between two groups defined by a clinicopathological feature. The median value of a gene of interest was applied as the threshold for the low and high expression groups of CC patients. The log-rank test and Kaplan-Meier survival curves were used to describe survival differences between two groups. All these analyses were conducted with GraphPad Prism 8.0.1.

## Results

### OPN Was Upregulated in CC and Correlated With Multiple Clinicopathological Parameters

**FIGURE 1:**
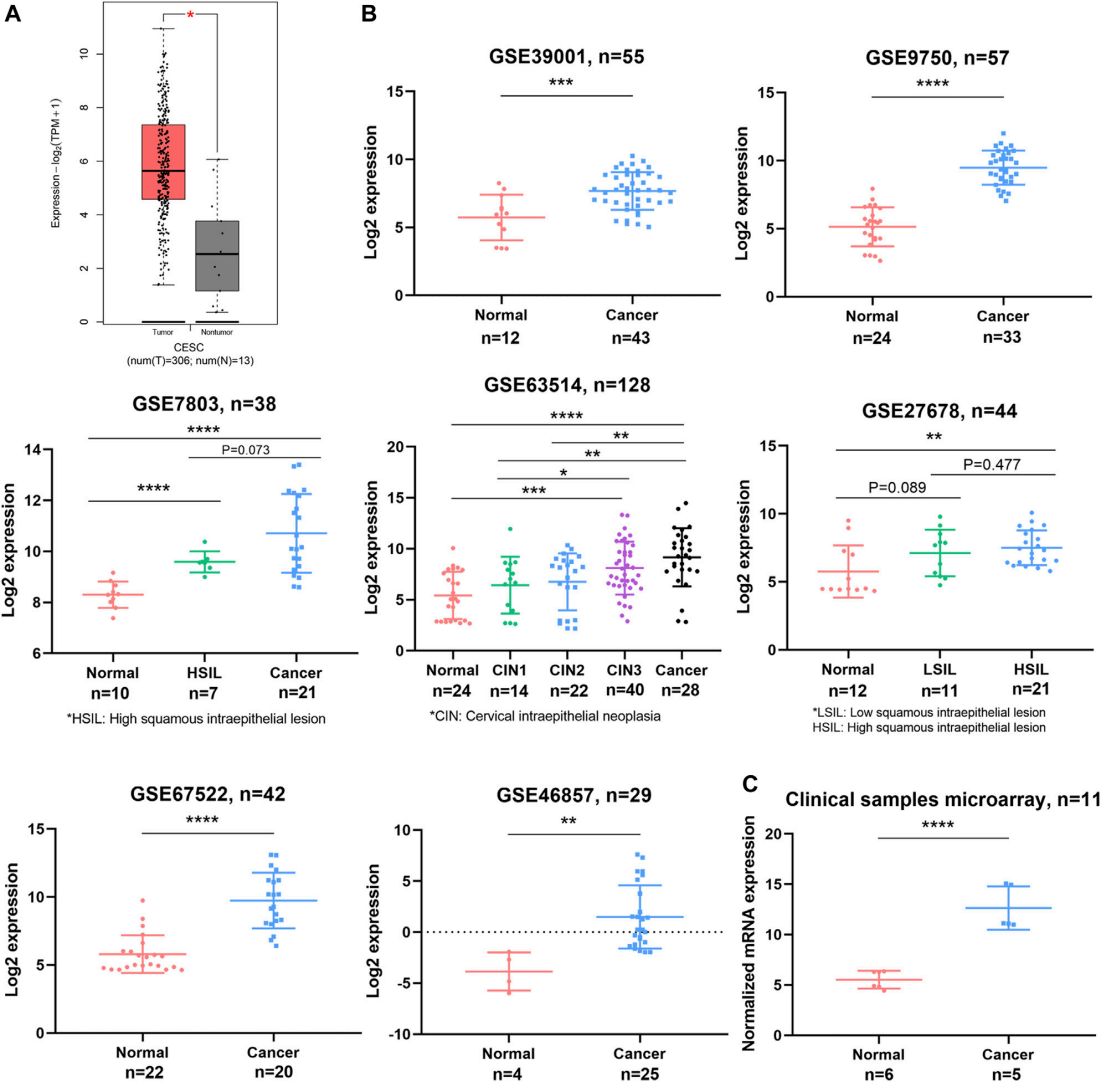
OPN expression was upregulated in CC tissues compared with normal tissues. Comparisons were conducted using data from the TCGA and GTEX databases **(A)**, GEO datasets **(B)**, and a clinical sample microarray **(C)**. *, *p*＜0.05; **, *p* < 0.01; ***, *p* < 0.001; ****, *p* < 0.0001.

According to GEPIA, higher expression of OPN was observed in CC samples than in nontumor samples (*p* < 0.05) (Figure 1A). For further validation, we investigated the difference in OPN expression between normal tissue and CC samples based on the GSE39001, GSE9750, GSE7803, GSE63514, GSE27678, GSE67522, and GSE46857 datasets and our clinical sample microarray (Figure 1B,C). All datasets revealed that OPN was upregulated in the cancerous tissue groups.

**FIGURE 2:**
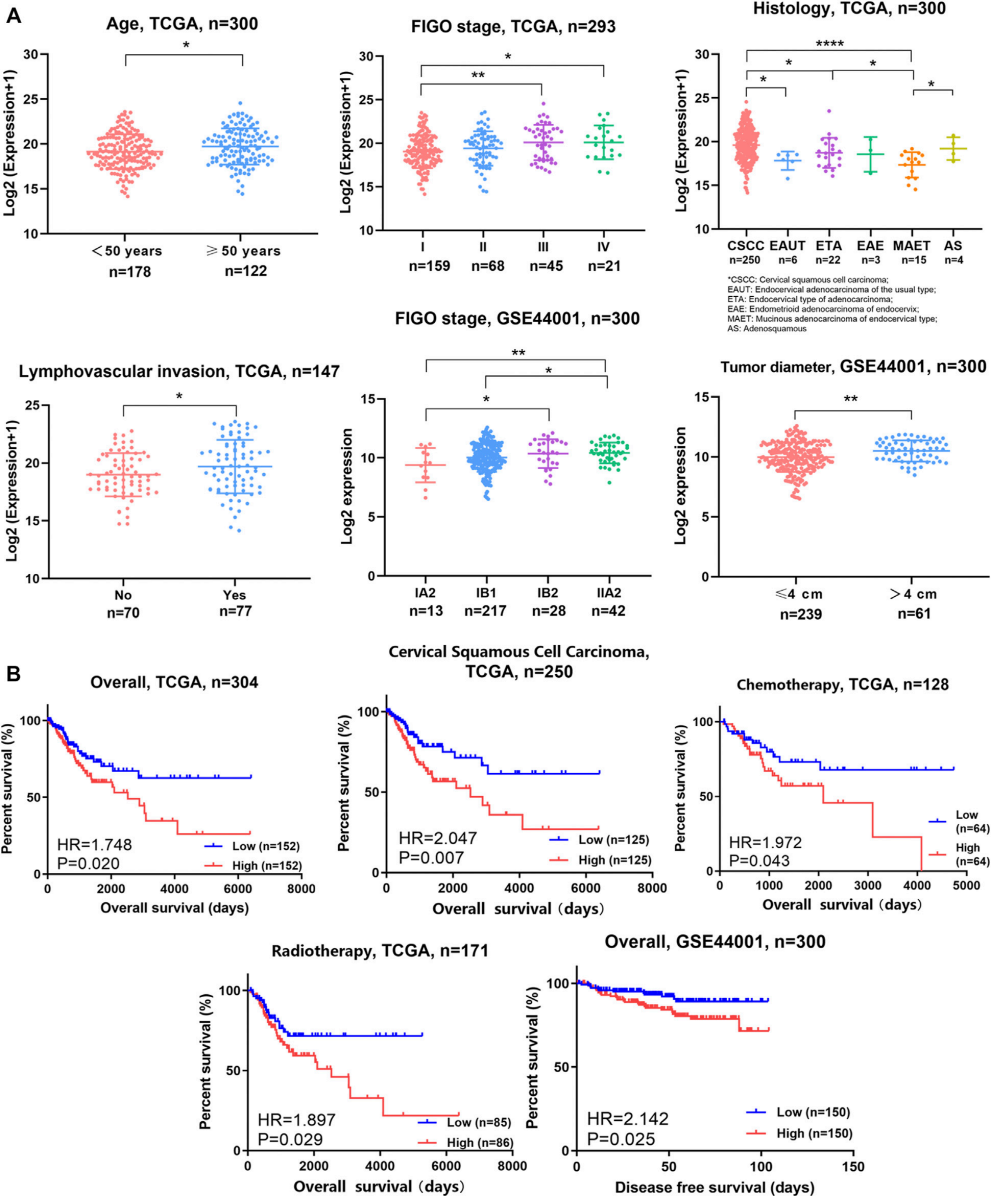
OPN expression was correlated with multiple clinicopathological features, unfavorable survival, and therapeutic resistance in CC **(A)** OPN expression was correlated with clinicopathological features in TCGA data and GSE44001 **(B)** High expression of OPN predicted unfavorable survival and therapeutic resistance in TCGA data and GSE44001. *, *p*＜0.05; **, *p* < 0.01; ***, *p* < 0.001; ****, *p* < 0.0001.

Furthermore, OPN expression was analyzed according to clinical parameters with data from the TCGA (Figure 2A, Supplementary Figure S1A). Higher expression of OPN was found in patients who were over 50 years old or who exhibited an advanced FIGO stage (stage III and IV), cervical squamous cell carcinoma, or lymphovascular invasion (*p*＜0.05). However, there were no significant correlations between OPN expression and body mass index (BMI), HPV status, neoplasm histologic grade, lymph node metastasis or corpus uteri involvement (Supplementary Figure S1A).

In addition, GSE44001 was analyzed (Figure 2A). OPN was more highly expressed in samples with an advanced FIGO stage (stage III and IV) or a larger tumor size (tumor diameter over 4 cm).

These results suggest that higher OPN expression indicates more advanced malignancy in CC.

### High expression of OPN was associated with poor survival and therapeutic resistance in CC

Survival analyses were conducted for the whole group or subgroups based on data from the TCGA (Figure 2B, Supplementary Figure S1B). Patients with CC exhibiting higher OPN expression had significantly shorter survival times than their counterparts in the overall cohort (hazard ratio (HR) = 1.748, *p* = 0.020). In addition, we plotted survival curves for cervical squamous cell carcinoma patients. The overall survival time was shorter in the cervical squamous cell carcinoma patients with high OPN expression than those with low expression (HR = 2.047, *p* = 0.007). There was no significant difference in the overall survival time according to OPN expression in the early-stage (FIGO IA1-IIA2), advanced-stage (FIGO IIB-IVB) cohort or adenocarcinoma patients (Supplementary Figure S1B). In addition, the patients with higher OPN expression had significantly shorter disease-free survival times than those with lower expression in the GSE44001 dataset (HR = 2.142, *p* = 0.025) (Figure 2B).

Moreover, survival analyses of TCGA subgroups defined by treatment were used to evaluate the effectiveness of well-accepted treatments. The CC patients with lower OPN expression showed better responses to chemotherapy and radiotherapy than those with higher expression (Chemotherapy: HR = 1.972, *p* = 0.043, Radiotherapy: HR = 1.897, *p* = 0.029) (Figure 2B), while there were no differences in patients who underwent radical hysterectomy or received targeted therapy (Supplementary Figure S1B). These results indicate that OPN may contribute to therapeutic resistance to chemotherapy and radiotherapy.

### High OPN expression was accompanied by an increased immunosuppressive status in CC

**FIGURE 3:**
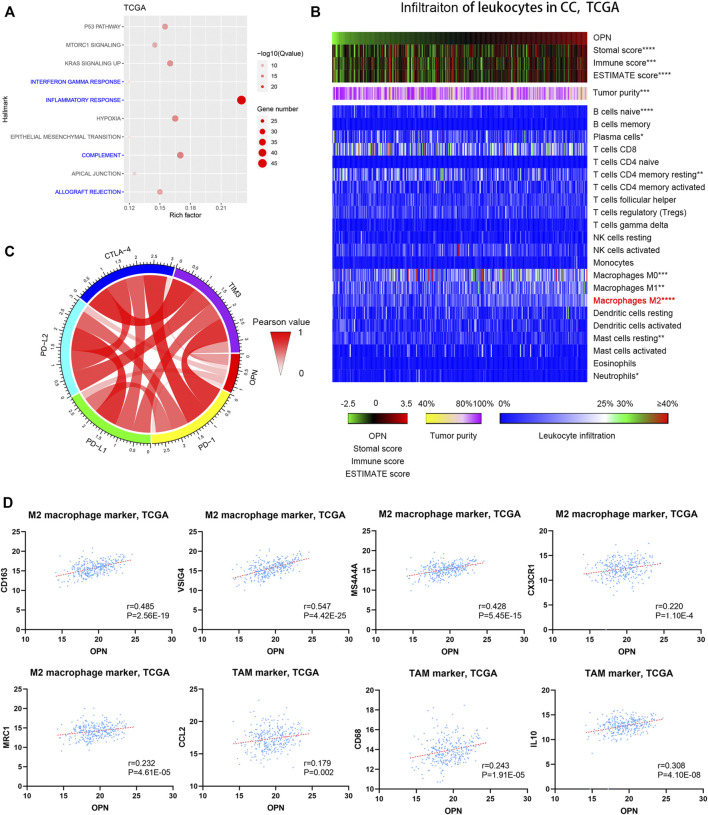
High OPN expression was accompanied by an increased immunosuppressive status in CC data from the TCGA **(A)** Hallmark enrichment analyses showed that OPN-associated genes were mainly enriched in biological processes of the immune response **(B)** The correlations between OPN expression and the infiltration of different kinds of leukocytes were calculated with Pearson correlation analysis and are shown in the heatmap. *, *p*＜0.05; **, *p* < 0.01; ***, *p* < 0.001; ****, *p* < 0.0001 **(C)** Circos plots show the correlations between OPN and five immune checkpoint molecules **(D)** Pearson correlation analysis showed that OPN was positively associated with M2 macrophage markers and TAM markers.

The biological function of OPN in CC has not been fully investigated. Therefore, we aimed to identify the possible biological function of OPN through analysis of the biological functions of OPN-associated genes. All the OPN-associated genes from the TCGA are listed in Supplementary Table S3 and were evaluated by Hallmark GSEA (Supplementary Table S4). The top 10 Hallmark terms identified by the Hallmark GSEA are listed in Figure 3A; the results indicated that OPN-associated genes were mainly enriched in biological processes of the immune response, including the inflammatory response, IFN-γ response, complement and allograft rejection (Figure 3A).

To evaluate the immunological status of CC, both tumor purity and the infiltration of 22 types of leukocytes were assessed for each sample. The samples are displayed in order of their OPN expression level. The results indicated that the immune score and the stromal score exhibited positive correlations with the OPN expression trends (Figure 3B, top panels), while tumor purity showed an inverse correlation with the OPN expression trends (Figure 3B, middle panels). Moreover, OPN expression was mostly related to the infiltration of M2 macrophages among the 22 types of leukocytes (Figure 3B, bottom panels). Pearson correlation analysis further showed that OPN was positively associated with M2 macrophage markers (CD163, VSIG4, MS4A4A, CX3CR1, and MRC1) and tumor-associated macrophage (TAM) markers (CCL2, CD68, and IL10) (Figure 3D).

To further explore the role of OPN in the immune microenvironment of CC, the correlations between OPN and five immune checkpoint molecules (PD-1, PD-L1, PD-L2, CTLA-4, and TIM-3) were analyzed (Figure 3C), which showed that OPN positively synergized with checkpoint molecules in CC. The results demonstrated that high OPN expression was accompanied by an increased immunosuppressive status.

Furthermore, the OPN-associated genes identified from GSE39001 and GSE9750 were also enriched in biological processes of the immune response (Supplementary Figure S2A). OPN was positively associated with some M2 macrophage markers (CD163, VSIG4, MS4A4A, and MRC1) and a TAM marker (CCL2) (Supplementary Figure S2B).

### High OPN expression was correlated with hypoxic conditions and high glycolytic levels in CC

**FIGURE 4:**
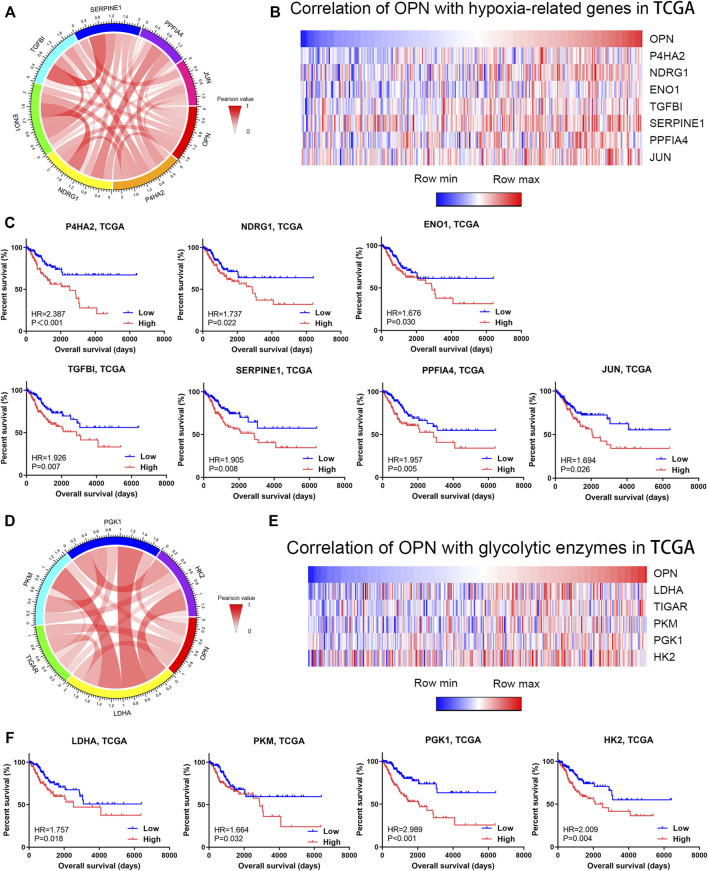
OPN was correlated with hypoxia and high glycolytic levels in CC data from the TCGA **(A,B)** Correlations of OPN with hypoxia-related genes **(C)** Survival plot for OPN-correlated hypoxia-related genes in CC patients **(D,E)** Correlations of OPN with glycolytic enzymes **(F)** Survival plot for OPN-correlated glycolytic enzymes in CC patients.

Hallmark GSEA of OPN-associated genes based on TCGA data showed that OPN was correlated with hypoxia (Figure 3A). As shown in the Circos plot and heatmap, OPN expression was positively correlated with multiple hypoxia biomarkers (P4HA2, NDRG1, ENO1, TGFBI, SERPINE1, PPFIA4 and JUN) (Figure 4A,B). Survival analysis was applied to these hypoxia-related genes, which showed that all these genes predicted an unfavorable prognosis in CC (Figure 4C). These findings indicated that OPN might be involved in CC progression induced by hypoxia.

To further investigate the metabolic role of OPN in CC, we analyzed the correlations between OPN and several glycolytic enzymes. A Circos plot and heatmap showed that the expression of OPN was positively correlated with that of glycolytic enzyme genes (LDHA, TIGAR, PKM, PGK1 and HK2) (Figure 4D,E). This indicated that higher OPN expression was correlated with a higher glycolytic level in CC. Except for TIGAR, the glycolytic enzyme genes predicted an unfavorable prognosis in CC (Figure 4F). These findings indicated that OPN might synergize with glycolytic enzymes in CC with a poor outcome.

### High OPN expression was correlated with apoptosis, angiogenesis, EMT, and multiple signaling pathways in CC

**FIGURE 5:**
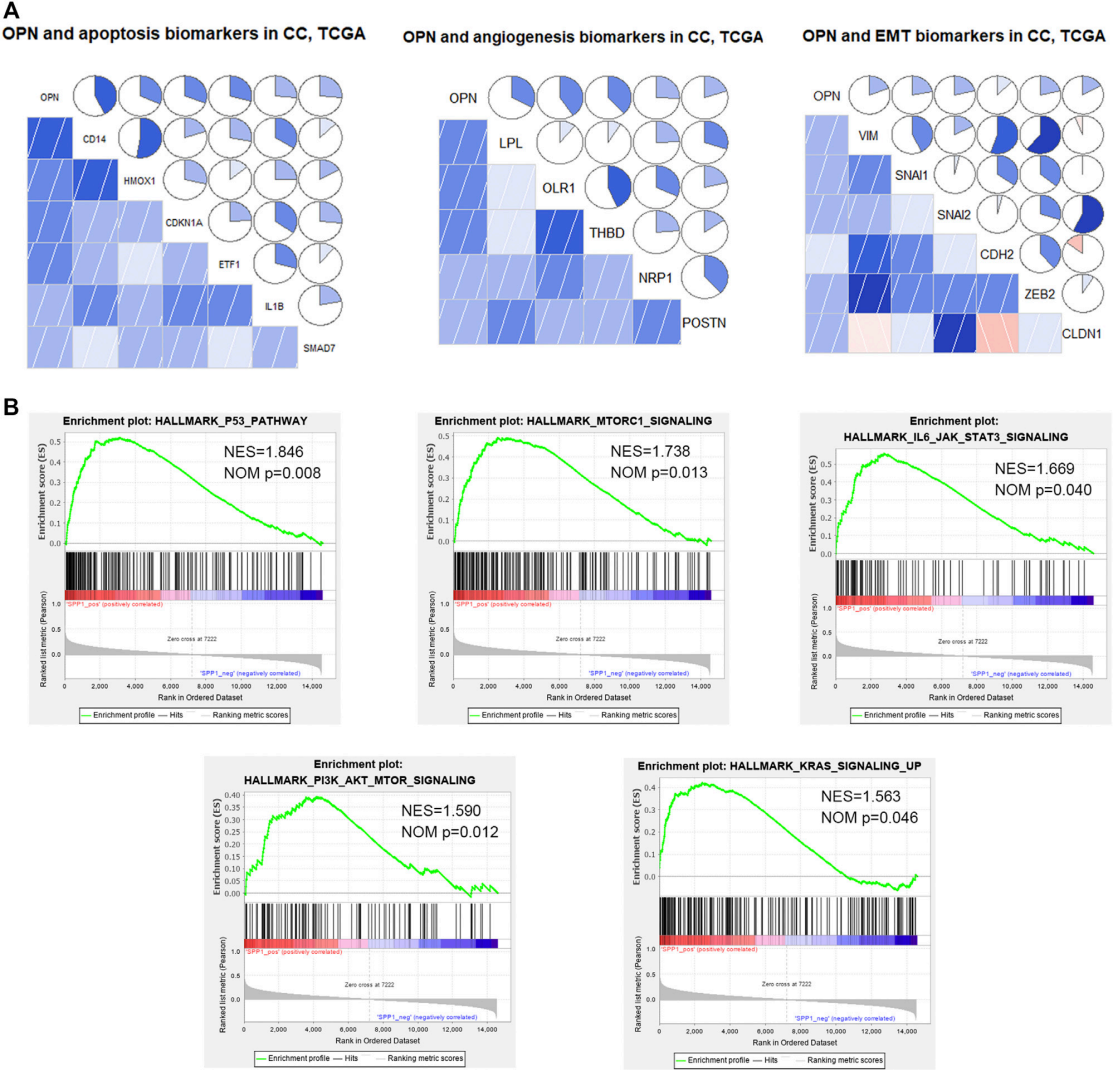
OPN was correlated with apoptosis, angiogenesis, epithelial-mesenchymal transition (EMT), and multiple signaling pathways in CC data from the TCGA **(A)** Relationships between OPN and apoptosis, angiogenesis, or EMT markers **(B)** GSEA showing the up-regulated signaling pathways enriched in OPN-associated genes.

According to hallmark GSEA results based on data from the TCGA, OPN was correlated with apoptosis, angiogenesis, and EMT in CC (Supplementary Table S4). Pearson correlation analysis showed that OPN was positively correlated with various apoptosis markers (CD14, HMOX1, CDKN1A, ETF1, IL1B and SMAD7), angiogenesis markers (LPL, OLR1, THBD, NRP1 and POSTN), and EMT markers (VIM/Vimentin, SNAI1/Snail, SNAI2/Slug, CDH2/N-cadherin, ZEB2 and CLDN1/Claudin-1) (Figure 5A, Supplementary Figure S3).

Moreover, GSEA of TCGA data showed that OPN was involved in several up-regulated signaling pathways, including the p53 pathway, IL6/STAT3 signaling, mTORC1 signaling, PI3K/Akt signaling and KRAS signaling (Figure 5B, Supplementary Table S5).

These results suggested that OPN might influence the tumor progression of CC through apoptosis, angiogenesis, EMT and the signaling pathways listed above.

## Discussion

OPN is a phosphorylated glycoprotein that bridges cell and matrix inorganic substances and is closely associated with the occurrence, progression and metastasis of malignant tumors (Le et al., 2003; Vergis et al., 2008; Raja et al., 2014; Ostheimer et al., 2017; Cao et al., 2019; Nishio et al., 2021). Recently, certain studies have indicated that OPN is a diagnostic and prognostic biomarker for CC (Cho et al., 2008; Leung et al., 2016). OPN expression was found to be positively related to FIGO stage, tumor size and cisplatin resistance (Cho et al., 2008; Chen et al., 2019). Moreover, high OPN expression was shown to predict poor overall survival and disease-free survival in CC patients (Vordermark et al., 2006; Manavi et al., 2007; Huang et al., 2015). By analyzing TCGA and GEO datasets and our tissue microarray, we found that OPN was more highly expressed in CC samples than in normal tissue samples. Additionally, OPN was significantly correlated with age, FIGO stage, tumor size, histology, lymphovascular invasion and poor outcomes. Moreover, CC patients with lower OPN expression showed better responses to chemotherapy and radiotherapy than those with higher expression in a subgroup survival analysis, which indicated that OPN might contribute to therapeutic resistance. All these results were consistent with previous reports. In conclusion, OPN was identified as a good diagnostic and prognostic biomarker for CC.

Only a few studies have investigated how OPN promotes CC progression. Vinit et al. found that OPN regulated the CD44-mediated p38 phosphorylation that induces NF-κB activation and the NF-κB–dependent expression of furin, an extracellular protease implicated in HPV processing that enhances CC cell motility (Kumar et al., 2010). Chen et al. found that OPN knockdown resulted in repressed proliferation and enhanced apoptosis in HeLa cells and that downregulation of OPN improved the cisplatin sensitivity of HeLa cells by inhibiting the PI3K/Akt signaling pathway (Vordermark et al., 2006; Manavi et al., 2007; Huang et al., 2015). Xu et al. found that overexpression of miR-181a could inhibit the expression of OPN, induce cell apoptosis, restrain cell proliferation, and reduce cisplatin resistance in CC cells (Vordermark et al., 2006; Manavi et al., 2007; Huang et al., 2015). In our study, we also found that OPN was positively correlated with apoptosis and the PI3K/Akt pathway. In addition, we found that OPN was involved in angiogenesis, EMT and several pathways including the p53 pathway, IL6/STAT3 signaling, mTORC1 signaling and KRAS signaling in CC. The roles of OPN in these pathways have been validated in other cancers (Vordermark et al., 2006; Manavi et al., 2007; Huang et al., 2015).

It is well established that EMT plays important roles in cancer progression, especially in tumor metastasis (Lee et al., 2008; Qureshi et al., 2015; Daugaard et al., 2017). Our previous studies confirmed the close relationship between EMT and CC metastasis (Shang et al., 2018; Liu et al., 2020; Zhang et al., 2020). Emerging reports have shown that OPN regulates the expression of EMT-related transcription factors, including Twist, Snail, Slug and zinc finger E-box-binding homeobox (ZEB), directly or indirectly in various cancers (Kothari et al., 2016). Herein, we showed that OPN was positively correlated with EMT biomarkers in CC, identifying a novel mechanism underlying CC metastasis.

To further investigate the function of OPN in CC, we explored the relationship between OPN and the tumor microenvironment in CC. As a main component of the tumor microenvironment, TAMs play an important role in CC. The polarization of TAMs toward an M2 phenotype is correlated with a poor prognosis, and M2 TAMs can transdifferentiate into lymphatic endothelial cells, inducing lymphangiogenesis and metastasis (Kerjaschki, 2005; Petrillo et al., 2015; Guo et al., 2016). Emerging studies have indicated that OPN induces M2 macrophage polarization, maintains M2 macrophage phenotypes, and acts as a chemoattractant for TAMs (Klement et al., 2021). We analyzed the correlations between OPN and the infiltration of all kinds of leukocytes. OPN expression was mostly related to the infiltration of M2 macrophages and positively related to M2 macrophage markers and TAM markers, indicating that OPN acts as an immune regulator in CC progression. OPN also acts as an immune checkpoint molecule to negatively regulate T cell activation (Kerjaschki, 2005; Petrillo et al., 2015; Guo et al., 2016). We found that OPN synergized well with immune checkpoint molecules (PD-1, PD-L1, PD-L2, CTLA-4, and TIM-3), further validating its immunosuppressive function. As treatments targeting immune checkpoints have shown initial success in CC (Kerjaschki, 2005; Petrillo et al., 2015; Guo et al., 2016), our study highlighted that OPN could be a novel target in immunotherapy.

As hypoxia and intensified glycolytic metabolism are characteristic features of solid tumors, including CC, we investigated the relationships between OPN and hypoxia or glycolysis in CC. Our study suggested that OPN was correlated with a hypoxic microenvironment. Previous reports showed that OPN was upregulated under hypoxic conditions in various cancers (Le et al., 2003; Vergis et al., 2008; Raja et al., 2014; Ostheimer et al., 2017; Cao et al., 2019; Nishio et al., 2021). Raja et al. showed that silencing OPN or its receptor significantly downregulated hypoxia-induced breast cancer cell migration and invasion and that HIF-1α was involved in this process (Le et al., 2003; Vergis et al., 2008; Raja et al., 2014; Ostheimer et al., 2017; Cao et al., 2019; Nishio et al., 2021). Yang et al. showed that hypoxic dendritic cells secreted large amounts of OPN, which were responsible for the enhanced migration of tumor cells (Yang et al., 2009). These findings indicate that OPN could be an important intermediate in hypoxia-driven CC progression.

In addition, our study suggested that high OPN expression was correlated with high glycolytic levels in CC. The relationship between OPN and glycolysis has been investigated previously in some cancers. Hsieh et al. showed that OPN regulated the expression of glucose transporter 1 and glucose transporter 3 in osteosarcoma and enhanced glucose uptake in cells via the integrin αvβ3 (Le et al., 2003; Vergis et al., 2008; Raja et al., 2014; Ostheimer et al., 2017; Cao et al., 2019; Nishio et al., 2021). Lu et al. showed that OPN enhanced hepatocellular carcinoma glycolysis by activating αvβ3-NF-κB signaling (Lu et al., 2020). Shi et al. reported that osteopontin-c, a splice variant of OPN, supported the anchorage independence of invasive breast cancer cells by utilizing glucose to generate energy (Le et al., 2003; Vergis et al., 2008; Raja et al., 2014; Ostheimer et al., 2017; Cao et al., 2019; Nishio et al., 2021). Therefore, OPN might upregulate glycolysis to generate energy to support the survival and expansion of CC cells.

## Conclusion

Our study is the first study to comprehensively explore the biological and clinicopathological characteristics of OPN in CC by using bioinformatics. We found that the expression of OPN was positively correlated with age, FIGO stage, tumor size, lymphovascular invasion and an unfavorable prognosis. Furthermore, we found that OPN was involved in immune suppression, hypoxia, high glycolytic metabolism, apoptosis, angiogenesis, EMT and multiple signaling pathways (p53 pathway, PI3K/Akt pathway, IL6/STAT3 signaling, mTORC1 signaling and KRAS signaling). Therefore, OPN represents a potential therapeutic target and useful prognostic factor in CC patients.

## Data Availability

The datasets presented in this study can be found in online repositories. The names of the repository/repositories and accession number(s) can be found in the article/Supplementary Material.
