# Supplementary figures and images for: Biological and Clinicopathological Characteristics of OPN in Cervical Cancers
Source: Front Genet. 2022 May 20;13:836509. doi: 10.3389/fgene.2022.836509 (PMC9163571; doi:10.3389/fgene.2022.836509)

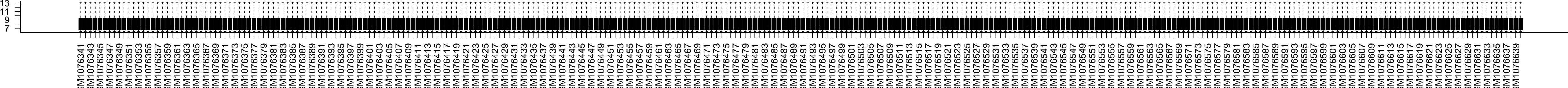

Supplement: Supplementary file 1 [file DataSheet2.PDF]

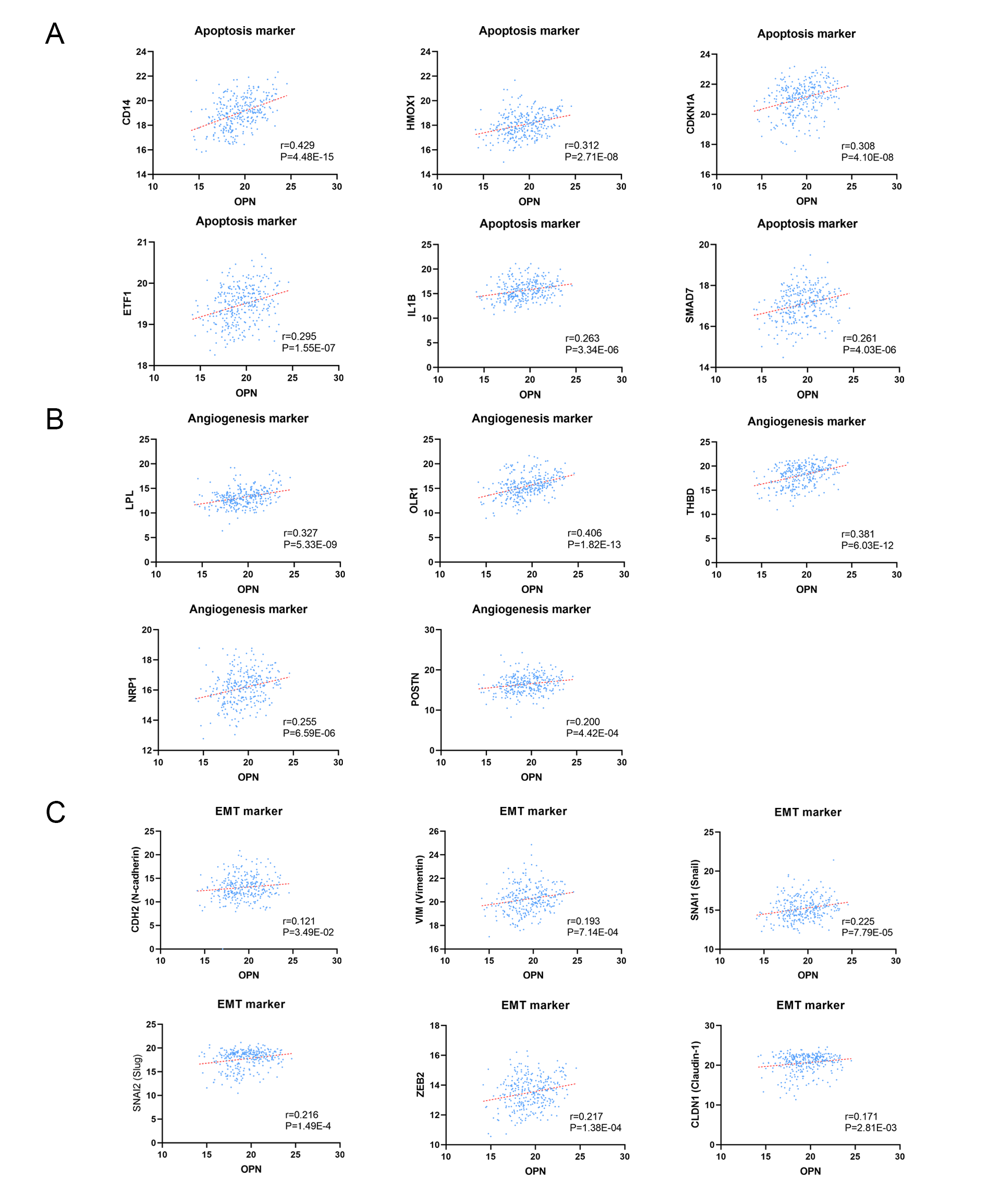

Supplement: Supplementary file 5 [file Image3.TIF]

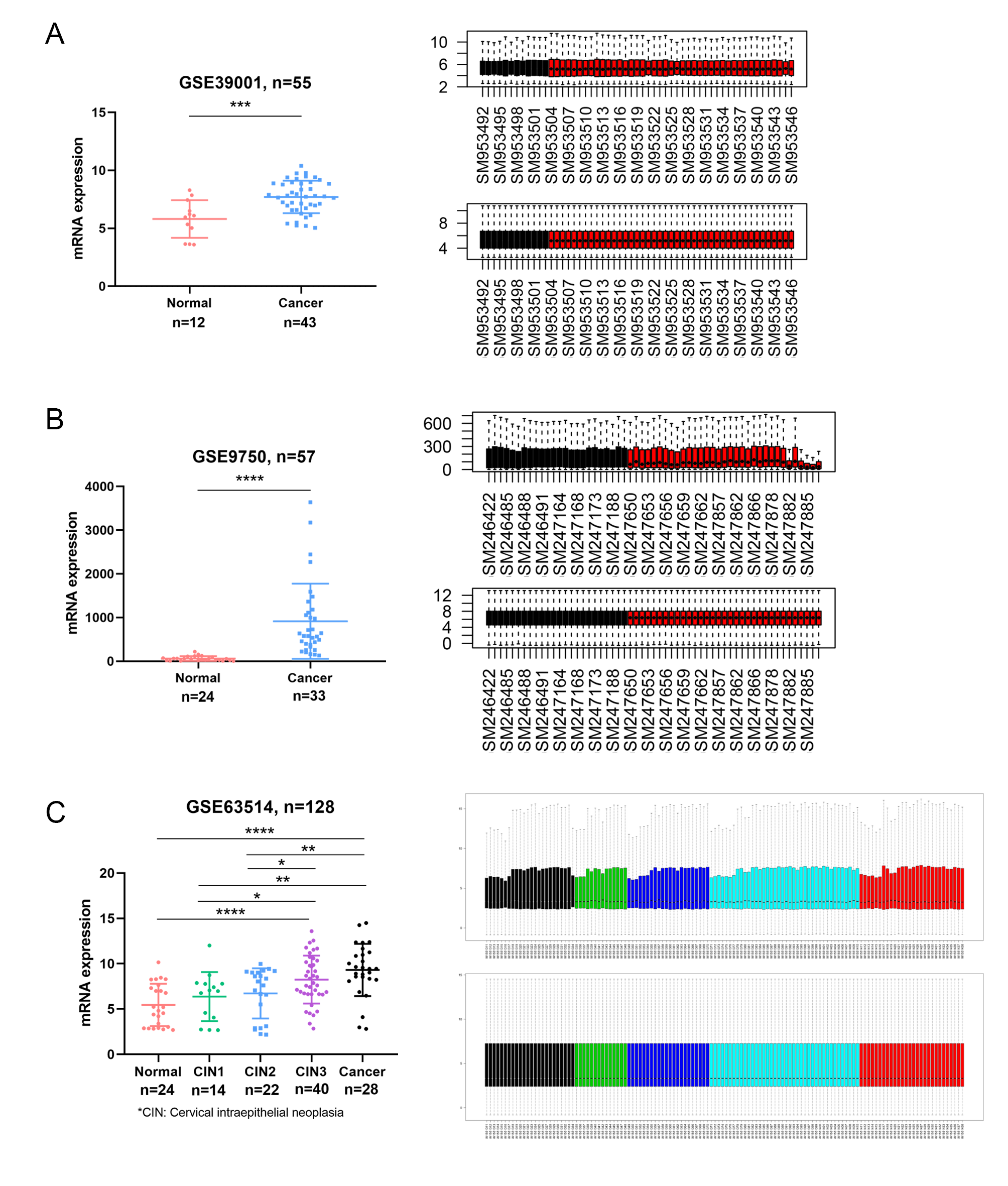

Supplement: Supplementary file 6 [file Image4.TIF]

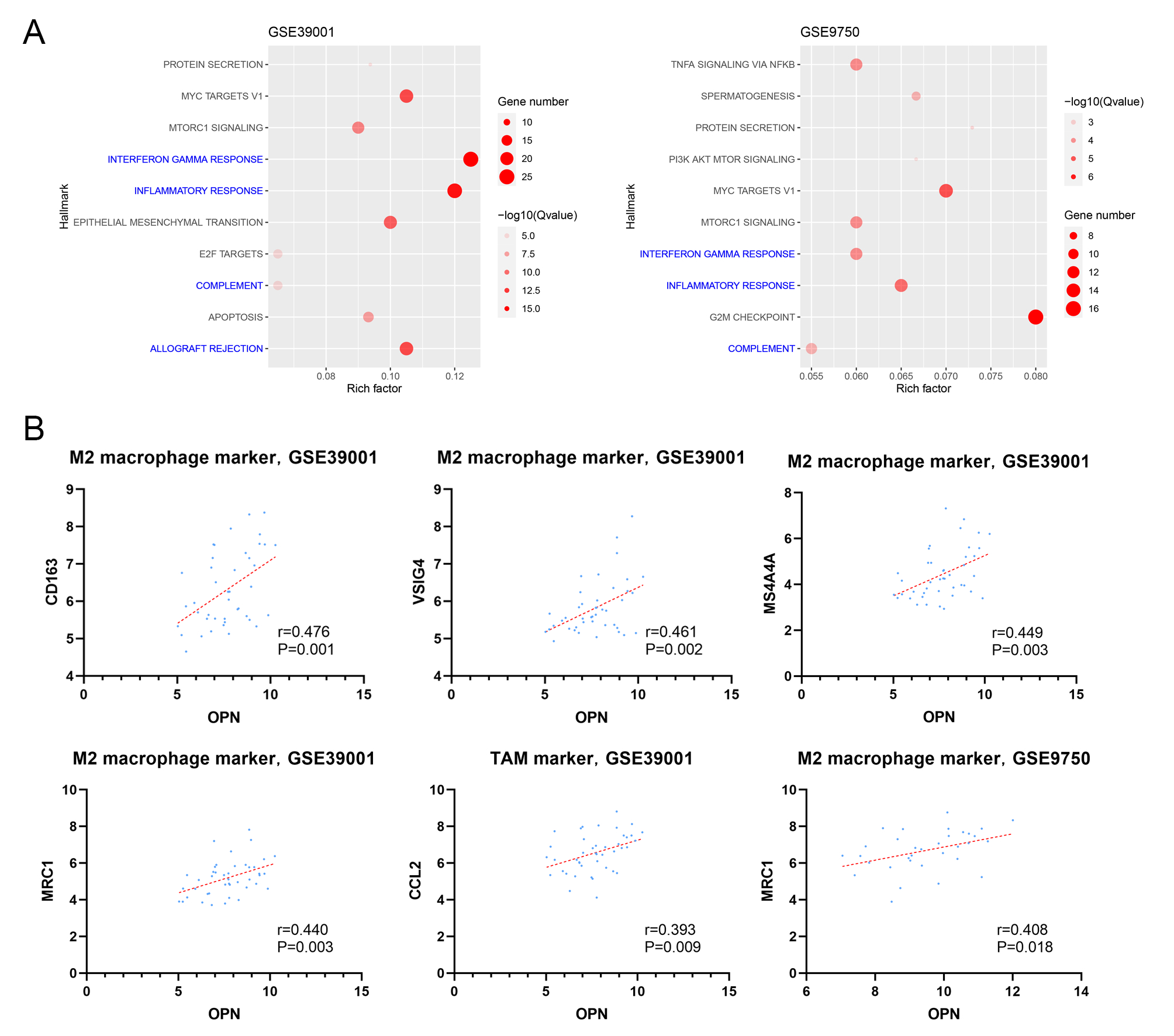

Supplement: Supplementary file 7 [file Image2.TIF]

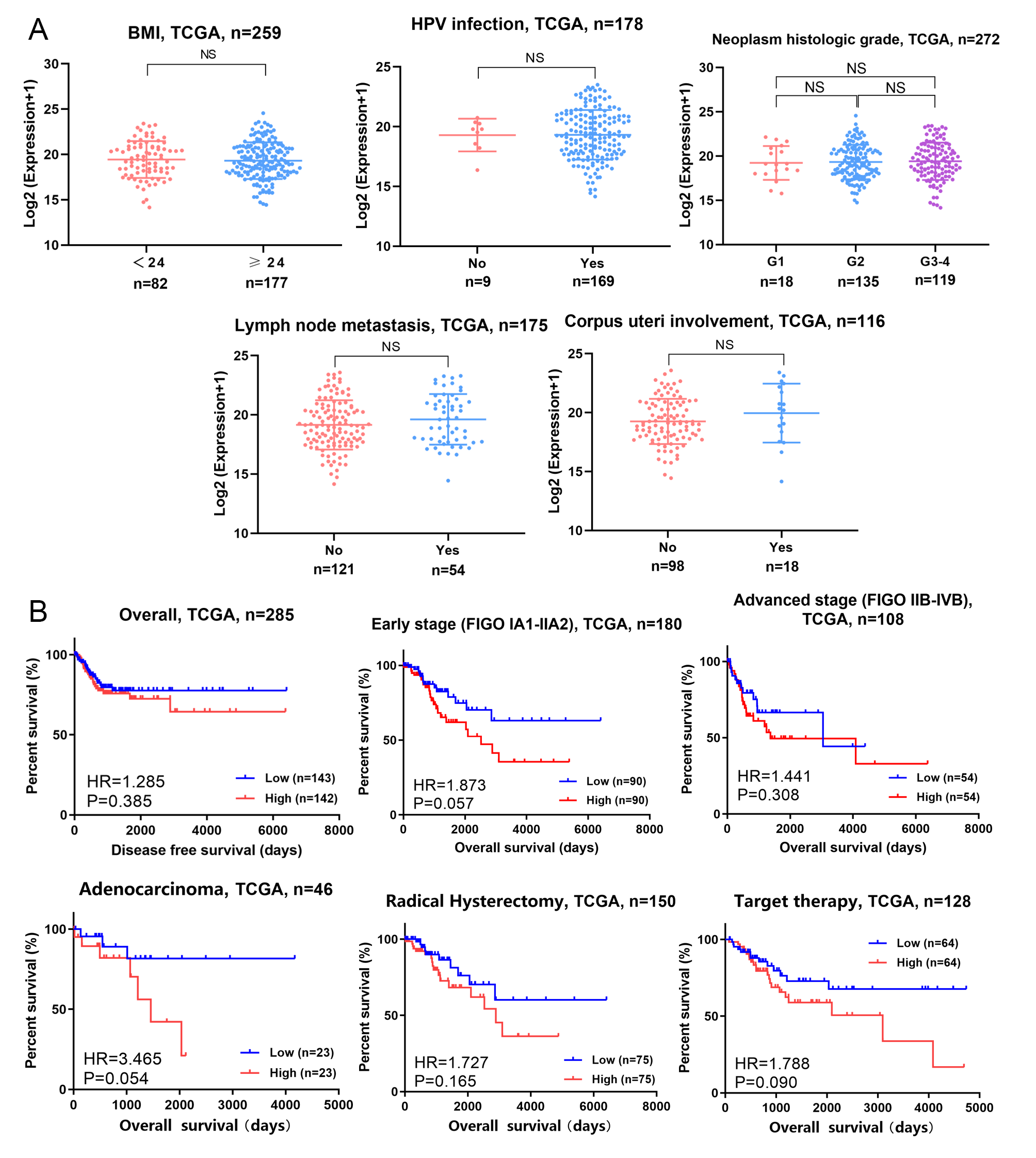

Supplement: Supplementary file 8 [file Image1.TIF]

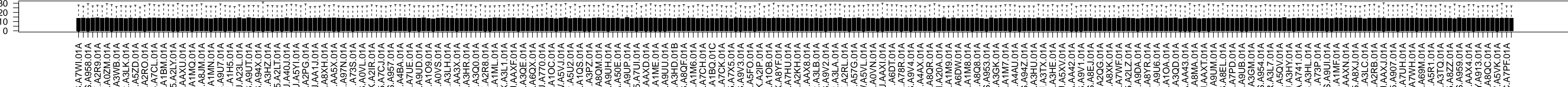

Supplement: Supplementary file 9 [file DataSheet3.PDF]

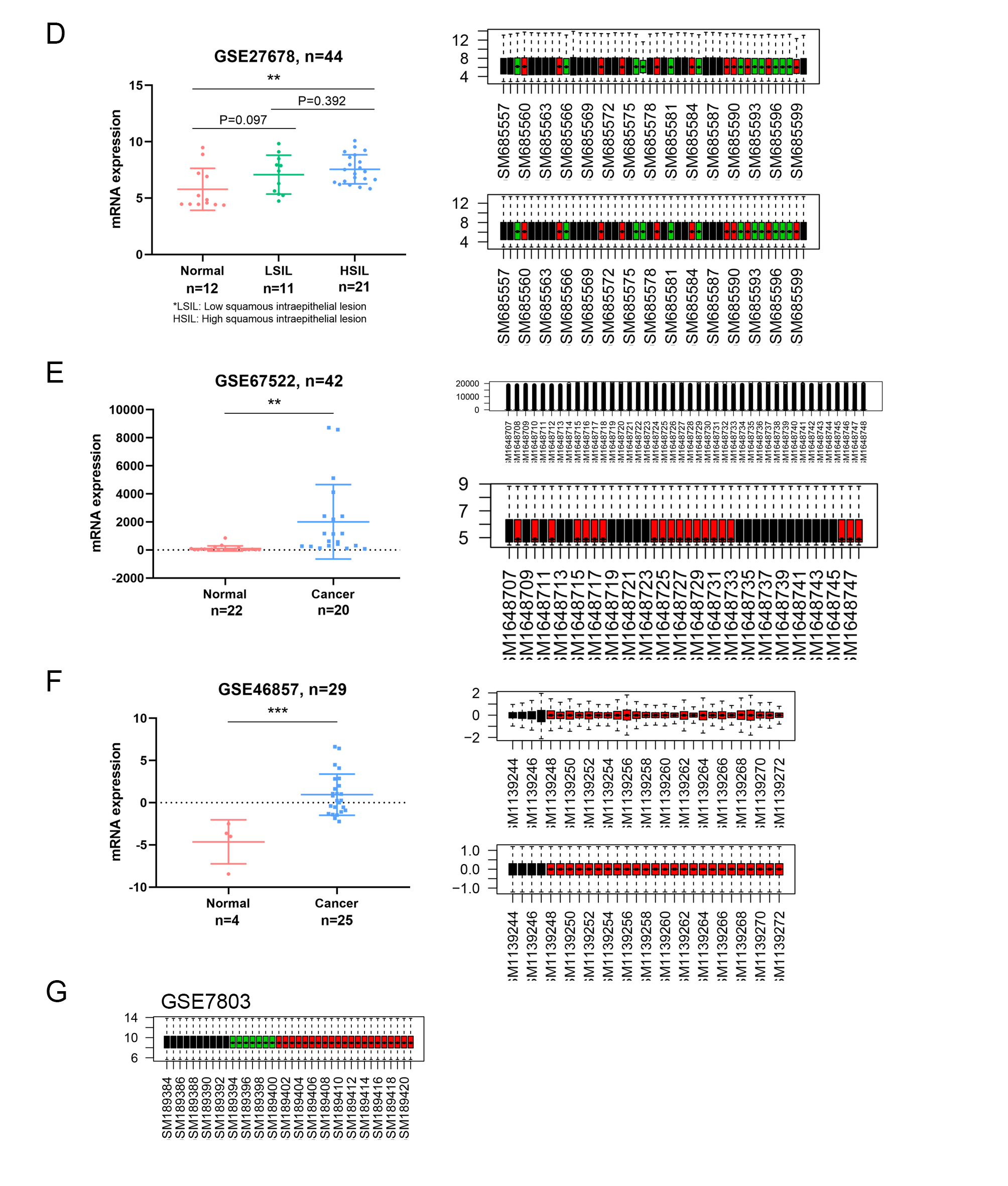

Supplement: Supplementary file 12 [file Image5.TIF]
