# Supplementary material for: Biological and Clinicopathological Characteristics of OPN in Cervical Cancers
Source: Front Genet. 2022 May 20;13:836509. doi: 10.3389/fgene.2022.836509 (PMC9163571; doi:10.3389/fgene.2022.836509)
Supplement: Supplementary file 4 [file Table1.DOCX]

Supplementary Material

# Supplementary Figures


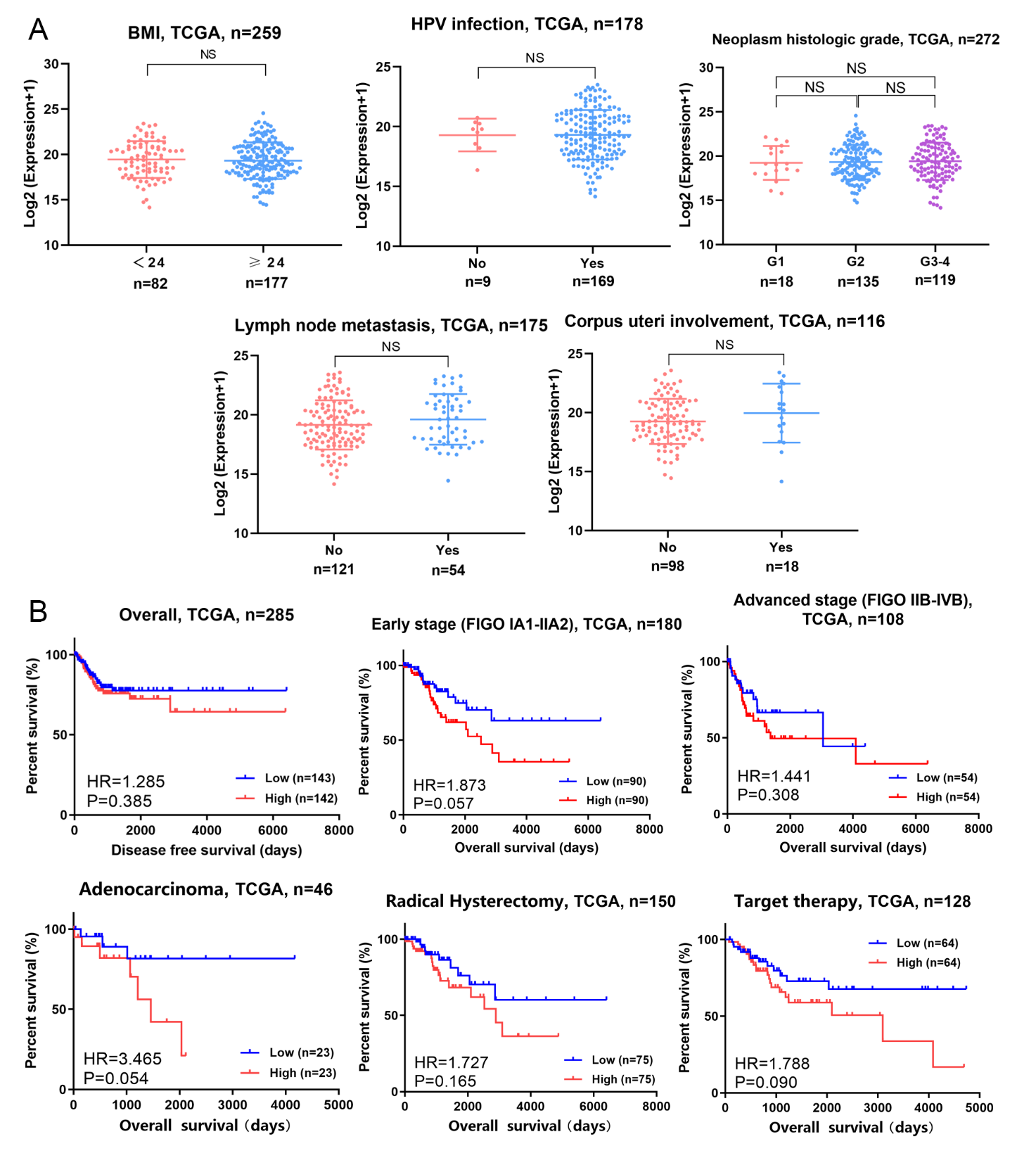


**Supplementary Figure 1.** Relationships between OPN expression and other clinicopathological features or the subgroup survival times in CC data from the TCGA. **(A)** Relationships between OPN expression and other clinicopathological features. **(B)** Subgroup survival analyses.


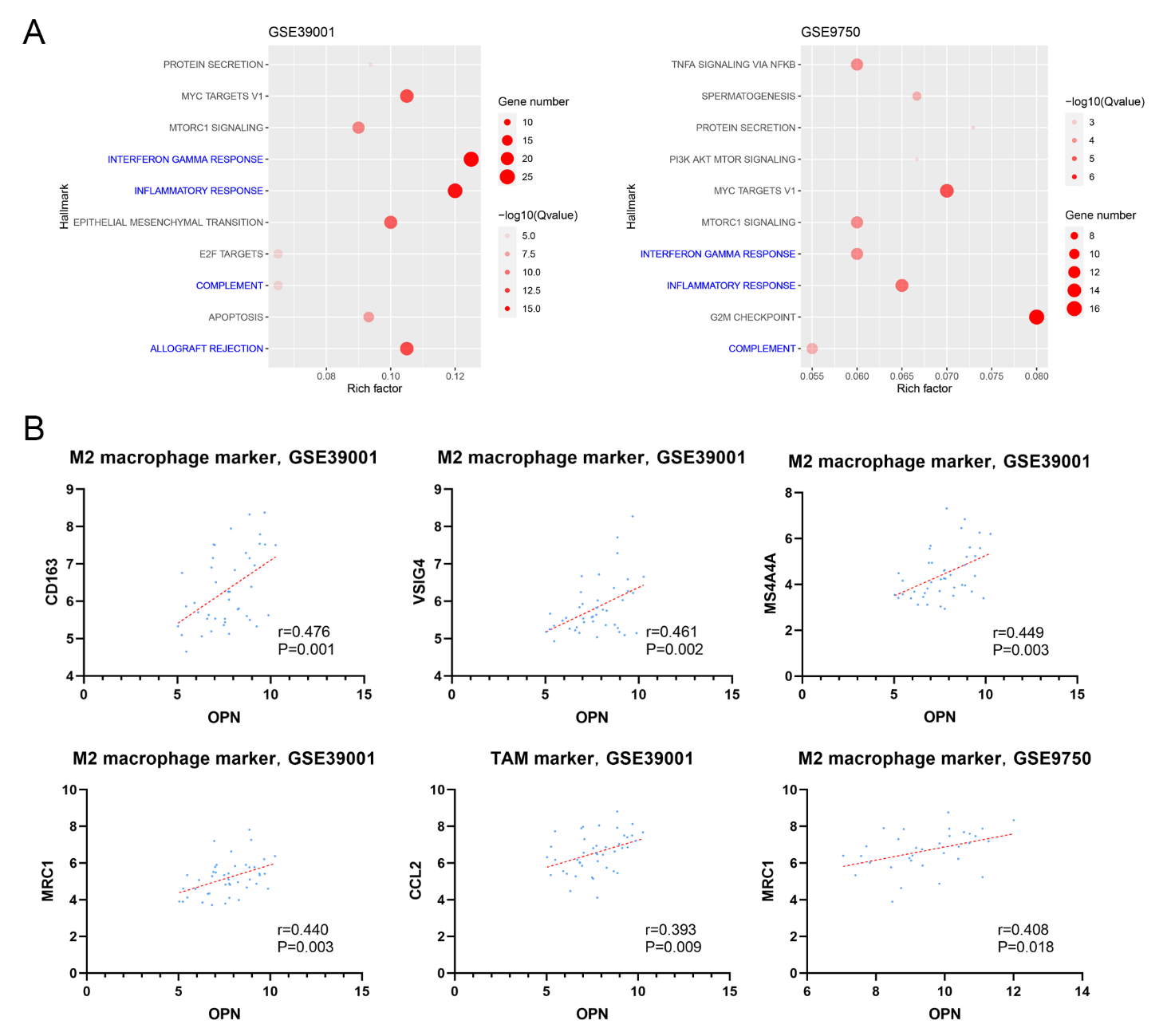


**Supplementary Figure 2.** High OPN expression in CC was accompanied by an increased immunosuppressive status in GSE39001 and GSE9750. **(A)** Hallmark enrichment analyses showed that OPN-associated genes were mainly enriched in biological processes of the immune response. **(B)** Pearson correlation analysis showed that OPN was positively associated with M2 macrophage markers and TAM markers.


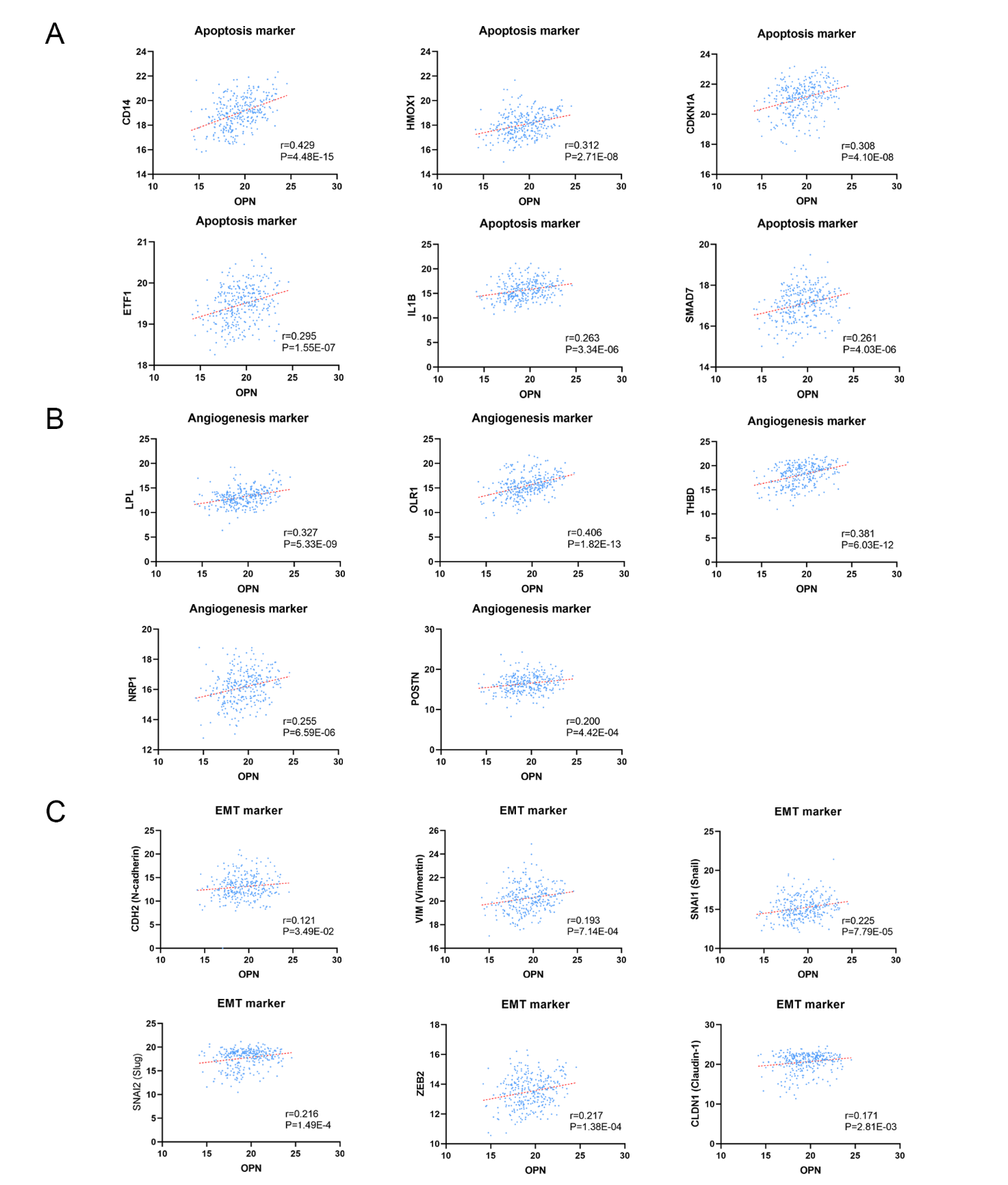


**Supplementary Figure 3.** OPN was correlated with apoptosis, angiogenesis, epithelial-mesenchymal transition (EMT) in CC data from the TCGA. Relationships between OPN and apoptosis **(A)**, angiogenesis **(B)**, or EMT markers **(C)**.


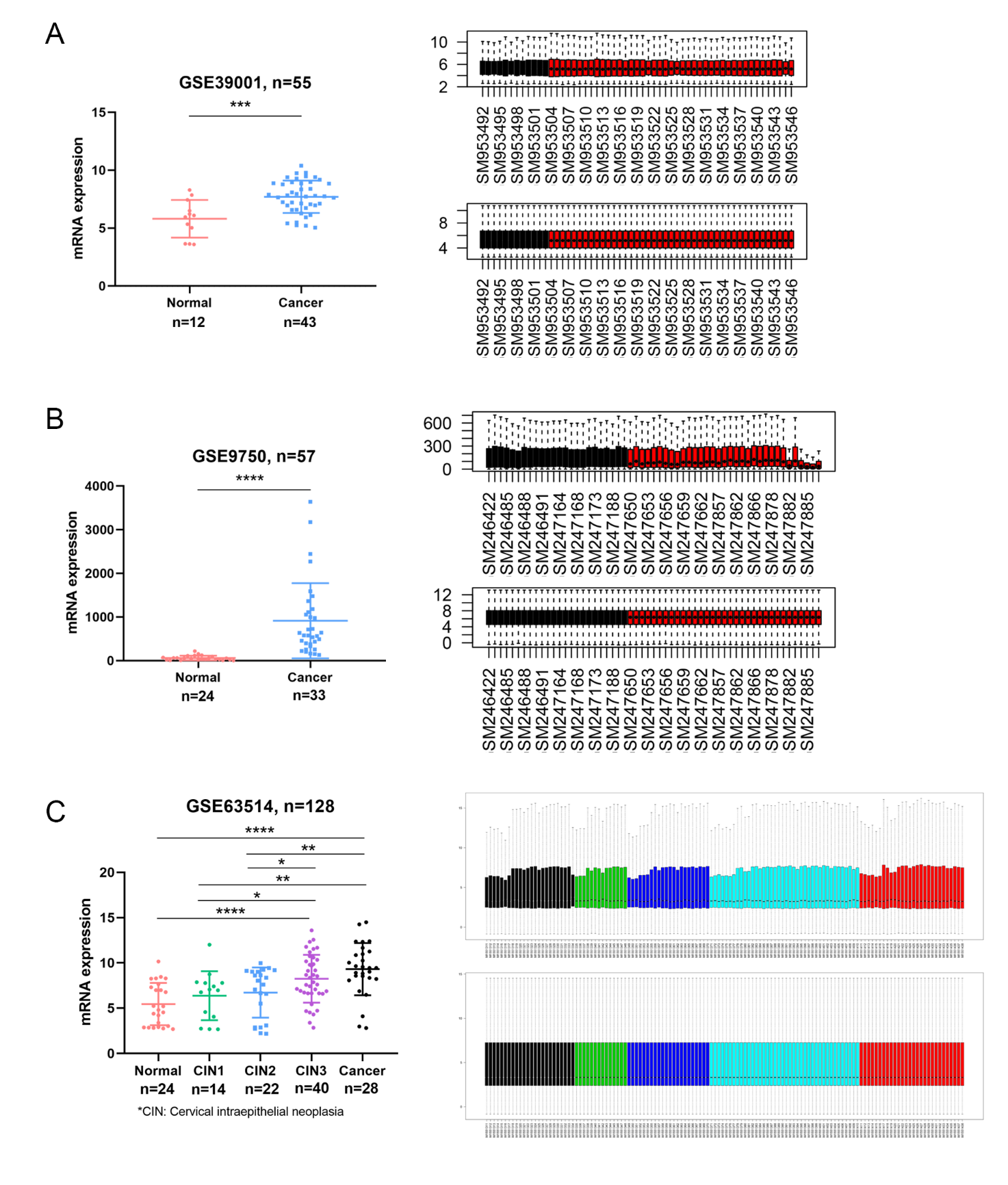


**Supplementary Figure 4.** The comparison diagram of OPN expression without normalization and Log2 transformation between CC tissues and normal tissues. The related boxplot of gene expression of samples from each GEO datasets before (top panels) and after (bottom panels) normalization and Log2 transformation. **(A)** GSE39001. **(B)** GSE9750. **(C)** GSE63514.


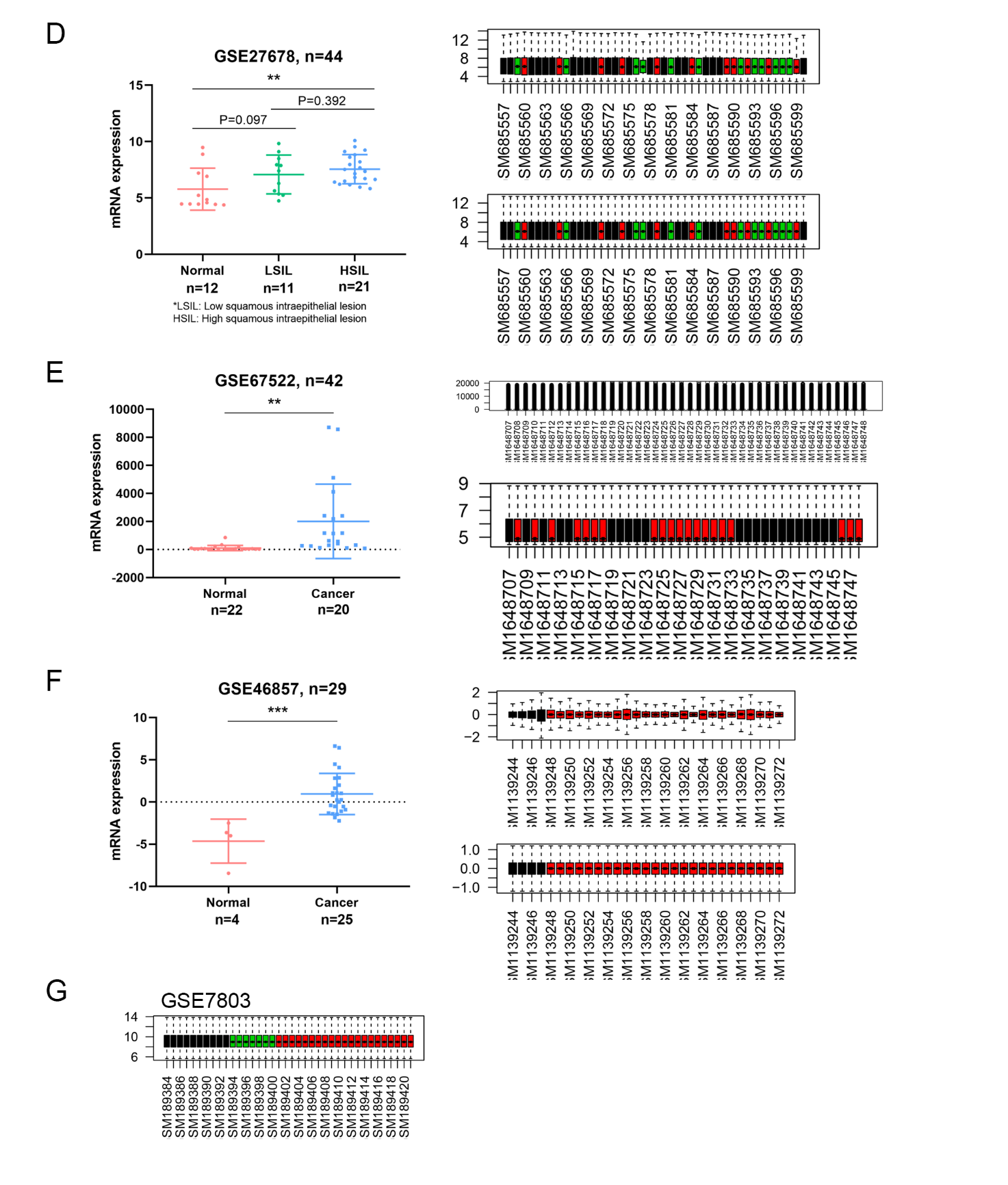


**Supplementary Figure 4 (continued).** The comparison diagram of OPN expression without normalization and Log2 transformation between CC tissues and normal tissues. The related boxplot of gene expression of samples from each GEO datasets before (top panels) and after (bottom panels) normalization and Log2 transformation. **(D)** GSE27678. **(E)** GSE67522. **(F)** GSE46857. **(G)** The boxplot of gene expression of samples from GSE7803 before normalization and Log2 transformation revealed the downloaded data had been standardized.

**Supplementary Figure 5** was showed in Supplementary data.

**Supplementary Figure 5.** The boxplot of gene expression of samples from GSE44001 before normalization and Log2 transformation revealed the downloaded data had been standardized **(A)**. The boxplot of gene expression of samples from TCGA revealed the downloaded data had been standardized **(B)**.

# Supplementary Tables

Showed in Supplementary data.
